# Risk of Drug-Drug Interactions in Out-Hospital Drug Dispensings in France: Results From the DRUG-Drug Interaction Prevalence Study

**DOI:** 10.3389/fphar.2019.00265

**Published:** 2019-03-22

**Authors:** Louis Létinier, Sébastien Cossin, Yohann Mansiaux, Mickaël Arnaud, Francesco Salvo, Julien Bezin, Frantz Thiessard, Antoine Pariente

**Affiliations:** ^1^Inserm, UMR 1219, Team Pharmacoepidemiology, Bordeaux Population Health Research Center, University of Bordeaux, Bordeaux, France; ^2^Service de Pharmacologie Médicale, Pôle de Santé Publique, CHU de Bordeaux, Bordeaux, France; ^3^Inserm, UMR 1219, Team ERIAS, Bordeaux Population Health Research Center, University of Bordeaux, Bordeaux, France; ^4^Service d’Information Médicale, Pôle de Santé Publique, CHU de Bordeaux, Bordeaux, France

**Keywords:** drug interactions, pharmacoepidemiology, medication errors, claim database, antiarrhythmic drugs

## Abstract

**Introduction:** Drug interactions could account for 1% of hospitalizations in the general population and 2–5% of hospital admissions in the elderly. However, few data are available on the drugs concerned and the potential severity of the interactions encountered. We thus first aimed to estimate the prevalence of dispensings including drugs Contraindicated or Discommended because of Interactions (CDI codispensings) and to identify the most frequently involved drug pairs. Second, we aimed to investigate whether the frequency of CDI codispensings appeared higher or lower than the expected for the drugs involved.

**Methods:** We carried out a study using a random sample of all drugs dispensings registered in a database of the French Health Insurance System between 2010 and 2015. The distribution of the drugs involved was described considering active principles, detailing the 20 most frequent ones for both contraindicated or discommended codispensings (DCs). To investigate whether the frequency of CDI codispensings appeared higher or lower than the expected for the drugs involved, we developed a specific indicator, the Drug-drug interaction prevalence study-score (DIPS-score), that compares for each drug pair the observed frequency of codispensing to its expected probability. The latter is determined considering the frequencies of dispensings of the individual drugs constituting a pair of interest.

**Results:** We analyzed 6,908,910 dispensings: 13,196 (0.2%) involved contraindicated codispensings (CCs), and 95,410 (1.4%) DCs. For CCS, the most frequently involved drug pair was “bisoprolol+flecainide” (*n* = 5,036); four out of five of the most represented pairs involved cardiovascular drugs. For DCS, the most frequently involved drug pair was “ramipril+spironolactone” (*n* = 4,741); all of the five most represented pairs involved cardiovascular drugs. The drug pair involved in the CC with the highest score value was “citalopram+hydroxyzine” (DIPS-score: 3.7; 2.9–4.6); that with the lowest score was “clarithromycin+simvastatin” (DIPS-score: 0.2; 0.2–0.3). DIPS-score median value was 0.4 for CCs and 0.6 for DCs.

**Conclusion:** This high prevalence of CDI codispensings enforces the need for further risk-prevention actions regarding drug-drug interactions (DDIs), especially for arrhythmogenic or anti-arrhythmic drugs. In this perspective, the DIPS-score we develop could ease identifying the interactions that are poorly considered by clinicians/pharmacists and targeting interventions.

## Introduction

Drug-drug interactions occur when the effects of one drug are modified by the concomitant use of a second drug (Magro et al., 2012). They constitute an important cause of adverse drug reactions that are mostly predictable and avoidable (Seymour and Routledge, 1998). Their prevention remains however complex in clinical practice as the number of drugs that can potentially interact is high. Additionally, the tools allowing to identify coprescriptions/codispensings at-risk for interactions appear difficult to use in clinical/officinal practice (van der Sijs et al., 2006; Payne et al., 2015; Zheng et al., 2018). Depending on studies, DDIs are estimated to cause 2–5% of hospital admissions in elderly patients (Becker et al., 2007; Olivier et al., 2009; Bénard-Laribière et al., 2015), and 1% of hospital admissions in the general population (Dechanont et al., 2014). Nowadays, the demographic and epidemiological transitions have led in a growing proportion of the population being aged and presenting with chronic comorbidities (Global Burden of Disease Study, 2015). This aging population is expected to result in an important use of drugs, and a high prevalence of polypharmacy and chronic polypharmacy (Haider et al., 2007; Nobili et al., 2011; Maher et al., 2014). Polypharmacy is defined by the World Health Organization as “the administration of many drugs at the same time or the administration of an excessive number of drugs” and continuous polypharmacy is limited to medications taken for prolonged and regular periods (Fincke et al., 2005). As the latter is the most important risk factor for DDIs, it is likely that the populational exposure to the risk conveyed by these deleterious drug associations will rise in the future, except if dedicated interventions succeed to constrain it (Guthrie et al., 2015; Stoll and Kopittke, 2015). If the overall impact and health burden represented by DDIs is difficult to assess given the wide heterogeneity of the adverse events they can induce (Lopez-Gonzalez et al., 2009; Aronson, 2015), the populational exposure to the risk of DDIs can conversely be estimated and characterized by identifying codispensings of drug pairs that are contraindicated or discommended, and by describing which of such drug pairs are frequently codispensed despite their concomitant use is advised against.

To date, most studies have investigated DDIs in an in-hospital setting, and little is not regarding the specificities of DDIs encountered in out-hospital prescriptions. Moreover, on the studies performed have mostly considered specific populations, either in terms of comorbidies, or countries (Tulner et al., 2008; Chatsisvili et al., 2010; Marzolini et al., 2010; van Leeuwen et al., 2013; Dirin et al., 2014). Data that could be extrapolated to the general out-hospital are thus very limited.; the main study we identified conducted from the general population estimates that 0.5% prescriptions contain a contraindicated drug combination, and 7% a clinically significant but controllable drug combination (Toivo et al., 2016).

At all ends, none of these intended to evaluate prescribers’ potential knowledge of interactions or their willingness to avoid associating contraindicated interacting drugs.

In this context, we first aimed to characterize the Contraindicated or Discommended because of Interactions (CDI codispensings) that could be identified from prescriptions performed in an out-hospital setting. Second, we aimed to investigate to what extent the frequencies of these CDIs could reflect their potential avoidance by prescribers.

## Materials and Methods

### Data Source and Study Population

This study was conducted using claims data from the EGB database, a 1/97th dynamic age- and sex-representative sample of the population covered by the French national healthcare system. Briefly, EGB contains individual and anonymous data for 670,000 persons on demographic data such as gender, age and dates of death. It also contains comprehensive data on outpatient drug dispensing, including the dose and the quantity of the drugs dispensed, the date of the dispensing and the characteristics of the prescriber (Bezin et al., 2017). No information is available in EGB concerning drugs that are not reimbursed by the French national healthcare system, inpatient drugs, and over-the-counter drugs.

In this study, a random sample of 100,000 patients who had received at least one drug dispensing was constituted for each quarter between 01/01/2010 and 31/12/2015. The randomization was carried out used random numbers generator. For each patient, all drug dispensed during the quarter of interest were considered. Thus, the same patient was able to contribute to several drug dispensings. These drug dispensings was defined as one to *n* drugs delivered by a pharmacist to a patient on the same day.

### Codispensings Contraindicated and Discommended Because of Interactions (CDI Codispensings)

Codispensings contraindicated and discommended because of interactions were determined using the national thesaurus elaborated by the French Medicines Agency (*Agence Nationale de Sécurité du Médicament et des produits de santé*, ANSM). This document entitled “*Thésaurus des interactions”* lists the DDIs that are considered clinically meaningful either on a drug class level or an individual drug level; its quality has been internationally recognized^1^. This thesaurus provides thus to health professionals an official source of information about the main clinically meaningful DDIs (i.e., about 50,000 pairs of drugs), with warnings on the risks they can convey and recommendations for their management. DDIs are classified into four categories: “to take into account,” “needing cautious use,” “discommended,” or “contraindicated.”

Although its PDF format makes it difficult to be used in routine practice by prescribers, the French thesaurus is often mentioned as a main source by clinical decision support systems. Moreover, this thesaurus constitutes an official and powerful tool for the study of the populational exposure to the risk of DDIs from medico-administrative databases.

To use this thesaurus we developed an R package to extract the text from the PDF file and structured its content into a more machine readable format (CSV)^2^. Because this thesaurus is updated every year we used the thesaurus version corresponding for each year. Using this thesaurus on our data set, we have identified, within all dispensings, DDIs associated to the highest level of risks for patients, i.e., those related to “*discommended*” and “*contraindicated*” drug concomitant uses. For each quarter, the prevalence of CCs and DCs was defined as the total number of drug pairs contraindicated or discommended among our patient sample. The proportion represented by a given drug pair within all identified contraindicated/discommended codispensing was estimated by reporting this number over the total number of dispensings in which a codispensing of contraindicated (or discommended) drugs has been identified.

### Statistical Analysis

The prevalence of dispensing and the corresponding 95% confidence interval was estimated for each pair of CDI codispensed drugs and for each quarter of analysis from the random sample. This prevalence was then extrapolated to the French population using the annual demographic data provided by the French National Institute for Statistics and Economic Studies (*Institut National de la Statistique et des Etudes Economiques*, INSEE)^3^. For this extrapolation, we have taken into account the proportion of drug users in the complete EGB, which is supposed to be representative of the general population.

A measure of association we denominated the DIPS-score was calculated for each pair of CDI codispensed drugs to determine whether these drugs are more commonly used together or not. The DIPS-score show if the prevalence of this pair was irregular considering the frequency of dispensing of the two drugs concerned. Considering the expected probability of a codispensing of two drugs as the product of the proportion of all dispensings represented by each of these drugs in our sample. The DIPS-score corresponded to the ratio between the observed proportion of codispensing of the drugs to this expected probability. In theory, CDI codispensed drugs should have a DIPS-score <1, as they are expected to be prescribed concomitantly much less frequently than might be expected with respect to their individual frequency of prescription. The 95% Confidence Interval (95% CI) was estimated using the formula allowing to compute a 95% CI for risk ratio under the hypothesis of a normal distribution^4^.

The list of the twenty CCs and DCs most frequently found over the entire study period and their corresponding DIPS-score was studied in detail.

Analyses were performed using SAS^®^ version 9.4 (SAS Institute Inc., Cary, NC, United States) and R version 3.2.3 (R Foundation for Statistical Computing, Vienna, Austria).

## Results

**Figure 1:**
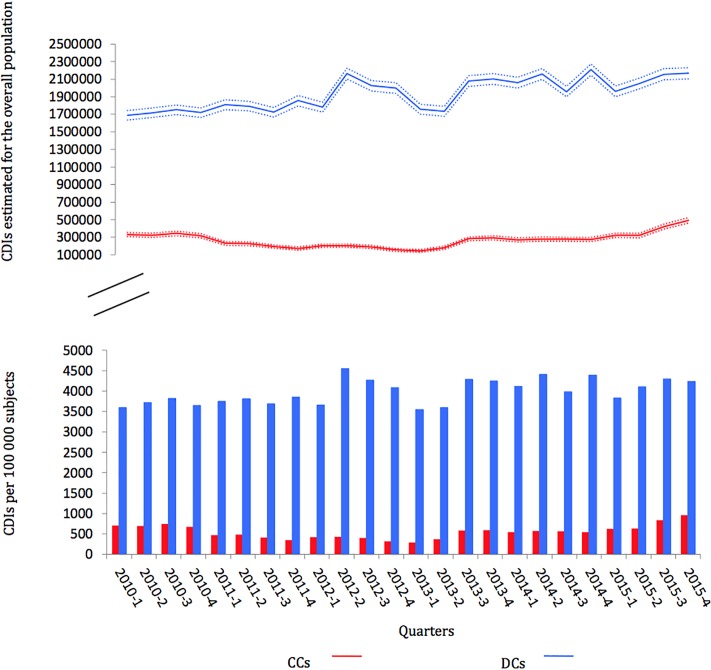
Number of observed contraindicated codispensings (CCs) and discommended codispensings (DCs) by quarter and extrapolation to the overall population with 95% CI.

Globally, the population considered in the random samples had a median age of 44.5 years (interquartile range: 24–63) and including 44.2% of male. A total of 6,908,910 drug dispensings was observed over the study period; among these, 13,196 implied CCs (0.2%; 95% CI: 0.2–0.2%) and 95,410 DCs (1.4%; 95% CI: 1.4–1.4%). Results by quarters over the study period and extrapolation to the overall French population are presented in Figure 1. According to these extrapolations, the number of CCs by quarter, rounded in thousands, fluctuated between 143,000 (95% CI: 126,000–160,000) and 490,000 (95% CI: 460,000–522,000), and the number of DCs by quarter fluctuates between 1,689,000 (95% CI: 1,635,000–1,744,000) and 2,210,000 (95% CI: 2,146,000–2,273,000).

**Figure 2:**
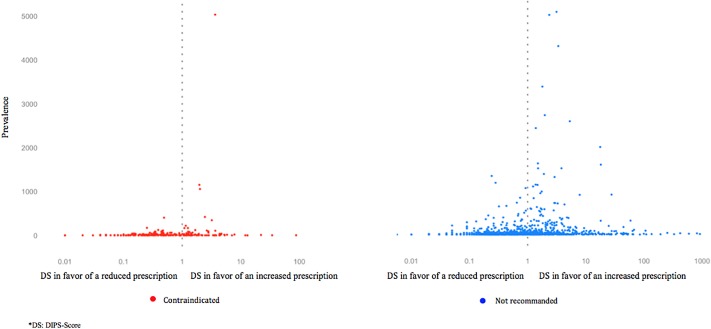
The 254 contraindicated and 1,111 discommended drug pairs by prevalence and DIPS-score.

Altogether, the different drug pairs involved in CDI codispensings consisted of 254 drug pairs for CCs and 1,111 drug pairs for DCs. The risk of CDI codispensing for each drug pair was studied using an *ad hoc* developed indicator. This indicator (DIPS-score) compares, for a given drug pair, the observed frequency of codispensing to its expected probability. The mean DIPS-score associated to CCs was 1.6 (median: 0.4; interquartile range: 0.2–1.3); it was 8.8 for DCs (median: 0.6; interquartile range: 0.2–1.8). Means exceeded medians because of extreme high values: DIPS-score max was 87.8 among contraindicated drug pairs and 2,700 among discommended pairs. These extreme values were found for drugs rarely prescribed, which limited the precision of DIPS-score estimations (Figure 2).

Table 1 shows the prevalence observed for the 20 drug pairs most frequently identified in CCs, together with the estimated value of the associated DIPS-score and its 95% CI. The drug pair involved in the most frequent CC was bisoprolol+flecainide for which 5,036 codispensings were identified, which corresponded to 38% of all identified CCs. The drug pair involved in the CC with the highest DIPS-score value was citalopram+hydroxyzine (3.7; 95% CI: 4.6–2.9); that associated with the lowest one was clarithromycin+simvastatin (0.2; 95% CI: 0.2–0.3).

**Table 1:** The twenty most frequently identified drug pairs involved in contraindicated codispensings over the study period.

		Prevalence	Proportion	DS^∗^
Drug A	Drug B	(*n* = 13,196)	(%)	(95% CI)
Bisoprolol	Flecainide	5,036	37.9	3.6 (3.4–3.9)
Potassium	Spironolactone	1,153	8.7	2.0 (1.8–2.2)
Flecainide	Nebivolol	1,058	8.0	2.0 (1.8–2.2)
Flecainide	Metoprolol	421	3.2	2.4 (2.0–2.9)
*Domperidone^∗∗^*	*Escitalopram*	*404*	*3.0*	*0.5 (0.4*–*0.6)*
Escitalopram	Hydroxyzine	346	2.6	3.2 (2.8–3.6)
Fluoxetine	Metoprolol	220	1.7	1.2 (1.0–1.4)
*Clarithromycin*	*Simvastatin*	*172*	*1.3*	*0.2 (0.2*–*0.3)*
Bisoprolol	Propafenone	168	1.3	1.3 (1.0–1.6)
Amiloride	Spironolactone	167	1.3	1.1 (0.9–1.4)
*Amiodarone*	*Domperidone*	*124*	*0.9*	*0.4 (0.3*–*0.5)*
Amiloride	Potassium	121	0.9	1.7 (1.2–2.2)
*Amiodarone*	*Cyamemazine*	*112*	*0.8*	*0.5 (0.4*–*0.6)*
Methotrexate	Trimethoprime	110	0.8	2.6 (1.8–3.8)
Citalopram	Hydroxyzine	106	0.8	3.7 (2.9–4.6)
*Citalopram*	*Domperidone*	*99*	*0.7*	*0.5 (0.4*–*0.6)*
Sotalol	Sulpiride	92	0.7	2.8 (1.9–4.1)
*Amiodarone*	*Sotalol*	*87*	*0.7*	*0.4 (0.3*–*0.5)*
Cyclosporine	Rosuvastatin	86	0.6	1.3 (0.9–1.7)
*Domperidone*	*Sotalol*	*85*	*0.6*	*0.3 (0.3*–*0.4)*

^∗^*DS, DIPS-score. ^∗∗^Drug pairs in italic correspond to those for which the observed prevalence is lower than expected (upper 95% CI < 1)*.

Table 2 shows the prevalence observed for the 20 most frequently identified drug pairs involved in DCs, together with the estimated value of the associated DIPS-score and its 95% CI. The drug pair involved in the most frequent DC was ramipril+spironolactone for which 4,741 codispensings were identified; this corresponded to 5% of all identified DCs. The drug pair involved in the DC with the highest DIPS-score was eplerenone+ramipril (18.1; 95% CI: 22.6–14.5); that associated with the lowest one was ibuprofen+ketoprofen (0.2; 95% CI: 0.2–0.3).

**Table 2:** The twenty most frequently identified drug pairs involved in discommended codispensings over the study period.

		Prevalence	Proportion	DS^∗^
Drug A	Drug B	(*n* = 95,410)	(%)	(95% CI)
Ramipril	Spironolactone	4,741	5.0	3.1 (3.0–3.3)
Perindopril	Spironolactone	4,676	4.9	2.4 (2.2–2.5)
Bisoprolol	Rilmenidine	4,012	4.2	3.4 (3.1–3.6)
Spironolactone	Valsartan	3,150	3.3	1.8 (1.7–1.9)
Candesartan	Spironolactone	2,542	2.7	2.0 (1.8–2.1)
Nebivolol	Rilmenidine	2,413	2.5	5.3 (4.8–5.9)
Irbesartan	Spironolactone	2,267	2.4	1.4 (1.3–1.5)
Valproic acid	Lamotrigine	1,863	1.9	17.7 (14.5–21.5)
Olmesartan	Spironolactone	1,514	1.6	1.5 (1.4–1.6)
Eplerenone	Ramipril	1,488	1.6	18.1 (14.5–22.6)
Cyamemazine	Escitalopram	1,410	1.5	3.8 (3.5–4.2)
Perindopril	Potassium	1,409	1.5	1.5 (1.4–1.6)
Losartan	Spironolactone	1,289	1.4	1.9 (1.7–2.1)
*Ibuprofen^∗∗^*	*Ketoprofen*	*1,247*	*1.3*	*0.2 (0.2*–*0.3)*
Enoxaparin	Ketoprofen	1,225	1.3	2.9 (2.6–3.2)
*Diclofenac*	*Ibuprofen*	*1,101*	*1.2*	*0.3 (0.3*–*0.3)*
Irbesartan	Potassium	1,062	1.1	1.4 (1.3–1.5)
Potassium	Ramipril	1,056	1.1	1.5 (1.3–1.6)
Potassium	Valsartan	1,023	1.1	1.2 (1.1–1.4)
*Ezetimibe*	*Fenofibrate*	*988*	*1.0*	*0.9 (0.8*–*1.0)*

^∗^*DS, DIPS-score.^∗∗^Drug pairs in italic correspond to those for which the observed prevalence is lower than expected (upper 95% CI < 1)*.

**Figure 3:**
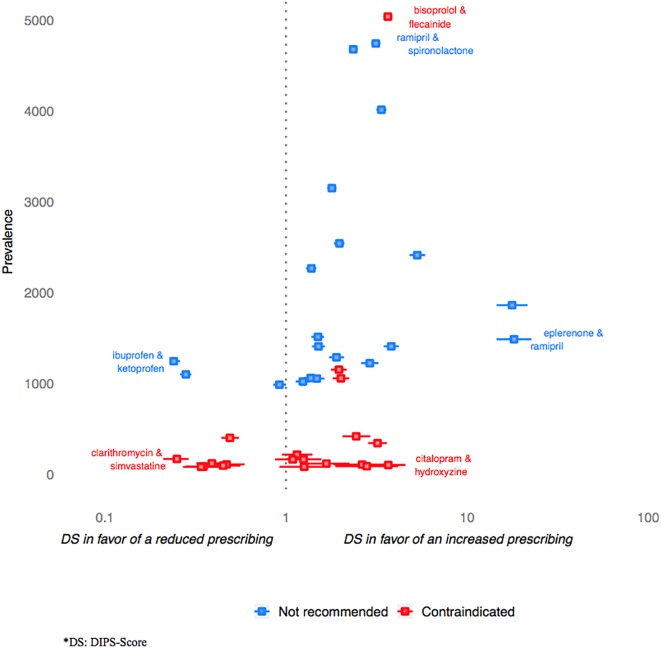
The twenty most frequent contraindicated and twenty most frequent not discommended drug pairs by prevalence and DIPS-score with 95% CI.

Distributions of the DIPS-score values for the 20 most frequent CCs and DCs are presented in Figure 3. For CCs, the prevalence was generally lower than for DCs, and the value of the associated DIPS-score more likely to be lower than 1. An exception was noted for the contraindicated “bisoprolol + flecainide” combination which presented with high values in terms of prevalence and DIPS-score value with regards to the distribution of this score for CCs (Table 1).

## Discussion

### Key Findings

According to our study, 140,000–500,000 dispensings of drugs would imply the concomitant delivery of contraindicated drugs each quarter in France; these numbers would be of 1,700,000 and 2,200,000 for discommended drug associations. The drugs mostly involved in CDIs were cardiovascular drugs with antiarrhythmic/arrhythmogenic properties and psychotropic drugs; the other identified involved drugs were very various. The important variations according to time depended both on changes in drug use and on changes in the listing of CDIs considered clinically relevant in the reference we used, and that is updated twice a year. However, overall, the global trend observed for the prevalence of these CDIs showed with an increasing trend from 2013 onward. If this can relate to the adding of new information on contraindications, this could also relate to the fact that the most concern drugs in age-related diseases, which use is thus supposed to increase with population aging. Yet, regarding at least contraindications, this number would be expected to be much lower and to correspond to rare situations of absolute necessity and therapeutic dead end. These appealing result supports the need to increase DDIs prevention and management. In this, they could help prioritizing the actions that would need to be undertaken regarding to the prevalence of codispensings highlighted for the different drug pairs and to the health risk conveyed by the potential DDIs they carry (Pirmohamed et al., 2004; Hines and Murphy, 2011; Montastruc et al., 2012).

Compared to other studied performed in the general population in an out-hospital settings, our results show with lower estimates of prevalence (Merlo et al., 2001; Zwart-van Rijkom et al., 2009; Toivo et al., 2016). Aside from the potential differences that can exist between countries, the main reason for these more conservative findings is that we restricted our study to CDIs considered to be clinically meaningful. In the study by Toivo et al. (2016) for instance, the authors used the FASS classification. This classification lists DDIs according to 4 levels of potential DDIs, and seems more extensive than the one we used but seems less selective than the French thesaurus. In particular the especially for level C DDIs (clinically significant but controllable drug combination) which corresponds to situations were “interaction may modify the effect of the drug, controllable, e.g., by dose adjustment”^5^.

If the issue is well-identified, it currently finds no simple answer aside of enforced education and information. Studies that focused on the interest of prescribing tools using clinical decision support system indeed showed that the tools evaluated were lacking of relevance for this specific objective (Nanji et al., 2017; Zenziper Straichman et al., 2017). One of the main criticisms emitted regarding these tools is related to their exhaustive consideration of each and any potential interaction. Indeed, in the prescription support they allow, interactions are treated equally whatever their clinical relevance, which results in excessive warning and, paradoxically, in alert fatigue and lack of efficacy (Payne et al., 2015).

The last important result we provided is the estimation of the statistical likelihood of the codispensings we performed considering the individual frequency of dispensing of each involved drug. This DIPS-score developed in this aim provided results intrinsically consistent: its median value was lower for CCs than for DCs (0.4 vs. 0.6) indicating that the first one were more likely to be avoided by physicians, and the proportion of CCs with a value of DIPS-score lower than 1 was much higher than that of DCs. By allowing ranking interacting drug pairs according to the potential unawareness or lack of perception of the risk associated with their codispensing, this tool could provide helpful information for the regulator.

Among the situations highlighted, some appeared of specific importance. This was especially true for CCs or DCs presenting with the higher prevalence, and for those presenting with a prevalence far exceeding that expected for the drug-pair (i.e., associated with high DIPS-score values).

The prevalence of co-administration of QT prolonging medications was high in our study, and found associated to elevated values of DIPS-score (e.g., 3.7 for citalopram+hydroxyzine). This could indicate that physicians ignore or disagree with the importance of the associated risk of ventricular arrhythmias including torsade de pointes and sudden death (Wenzel-Seifert et al., 2011; Schlit et al., 2017). Indeed, aside of this risk, the co-prescription of citalopram and hydroxyzine appears consistent on a therapeutical basis. It constitutes a combination of one antihistaminic indicated in the treatment of anxiety to an antidepressant which would appear rational in the context of the management of patients with anxio-depressive disorders; there is however no specific recommendation advocating for the concomitant use of these drugs especially. If proven of real interest, the association would at least deserve a close electrocardiographic monitoring of QT in patients at the time each drug is initiated. If not, it would deserve a clear action of communication owing to the nature of the hazard risked and to the observed frequency of the co-delivery. The importance of the prevalence of this coprescription at least advocates for the elaboration of a guideline either recommending not to use these drugs in combination for the management of anxio-depressive disorders, either to do it with a specific electrocardiographic monitoring of QT that would need to be determined. This appears of specific importance as Schlit et al., among others, showed that concomitant treatment with drugs known to induce arrhythmia was the greatest risk factor for QT prolonging by hydroxyzine (Schlit et al., 2017).

Conversely, the results we found were in favor of a good knowledge by prescribers of the DDI concerning clarithromycin and simvastatin. This association is contraindicated as leading to an increase in statin serum concentration and to a consecutive rise in the risk of presenting with cytolytic hepatitis or rhabdomyolysis (Page and Yee, 2014). For this pair constituted of drugs frequently used in the population, the overall amount of co-deliveries was high. However, it should have been much higher owing to the individual frequencies of dispensings of these drugs. Indeed, the DIPS-score estimated for the pair was of 0.25, revealing the prevalence of the co-delivery was approximately four times lower than expected. Interestingly, both clarithromycin and simvastatin are named in full in the interaction section of these drugs summary of product characteristics. The fact that the interaction is well-identified conversely to other for which interactions are specified using drug class instead of individual drug names should be considered.

### Limitations and Strengths

Amongst the drug-pairs for which results deserve a special attention, those concerning beta-blocker and other antiarrhythmic agents need to be discussed, as they can be specifically affected by the limitations of the study we present. Our estimations are based on an automated analysis of the codispensings observed in the study population, disregarding indication or patient background. As bisoprolol, for instance, is contraindicated with class one antiarrhythmic agents (e.g., flecainide) only in patients with cardiac failure, these results would need to be further explored. Contraindicated beta-blocker and antiarrhythmic agents were indeed found to be by far the most prevalent CCs, and to be coprescribed much more than expected. Interestingly in the last version of the Thesaurus, that was released in May 2018, these codispensings were no longer considered contraindicated but only discommended.

Among DCs, the drug pairs most frequently found were “ramipril+spironolactone” (5% of DCs) and “perindopril+spironolatone” (4.9% of DCs). If these codispensings are discommended to avoid hyperkalemia, their prevalence cannot be thought to represent the ignorance of the risk they carry. Indeed, it is likely that some patients in which these drugs are associated benefit from a close monitoring of kalemia. This was not evaluated in our study that only considered dispensings data.

Our objective was to identify CCs and DCs that would potentially result from a lack of knowledge of the risk conveyed, in order to help targeting future information campaigns/interventions aiming to minimize the risk associated to CDI codispensings. In this aim, we only considered co-prescriptions, and the proxy of such constituted by co-deliveries. The result herein presented thus correspond to an underestimated prevalence of concomitant use of drugs Contraindicated or Discommended because of Interactions, as they do not consider concomitant treatments that would result from successive but overlapping dispensings. If willing to estimate this overall prevalence, it would be needed to individualize sequences and overlaps of dispensing in patients, whatever the date of delivery. Additionally, as dealing with a reimbursement database, OTC deliveries cannot be considered for the estimation of the prevalence of concomitant treatments for contraindicated or discommended drug pairs. This is another factor of underestimation for the prevalence of use of drugs Contraindicated or Discommended because of Interactions, that however also not relate to a potential ignorance of the associated risk by physicians.

A specific strength of this study was to use a probabilistic method to allow evaluating to what extent drugs associations could be though or avoided. If allowing thus to assess the burden represented by each drug pairs amongst all situations at risk of potential interactions, and those that could be reduced, this study did not provide information on the adverse events actually engendered. In this perspective, it would be adequately completed by a study investigating the adverse reactions reported for the drug pairs herein identified. It would also be completed by studies investigating whether DDIs were ignored or deliberately overridden and considered to carry no or very weak risk with regard to the expected benefits.

The question raised by this work advocates for further development in studying CDIs in an out-hospital setting. DDIs are always considered according to situations of use placing the patient at-risk for an event, in the case the DDI would actually result in a clinically meaningful modification of the drug effect. To which extent the observed CDIs translate into clinical events that impact on patients and public health is not yet clearly quantified and should be investigated. Moreover, as this impact, at least in terms of population health, would both clearly depend on the frequency of exposure and on the baseline risk or frailties of the exposed population, studying it especially in the elderly would be of primary importance.

## Conclusion

The prevalence of codispensings of drugs Contraindicated or Discommended because of Interactions was found high. It concerned a large amount of drug-pairs; if these were heterogeneous, arrhythmogenic or antiarrhythmic drugs where largely represented. Despite these results did not consider the clinical consequences of these drug combinations, they provide a conservative but credible estimate of the potential iatrogenic burden of the DDIs as only codispensings related to clinically meaningful DDIs were considered. Together with this prevalence estimate allowing identifying which drug pairs were the most often encountered amongst these codispensings at-risk, we provided a tool, the DIPS-score, that could help identifying which DDIs might be the most ignored by prescribers and, potentially, identifying practices corresponding to new use of drugs.

## Author Contributions

All authors wrote the article. LL, SC, YM, JB, and AP designed the study. LL, SC, YM, JB, MA, and AP analyzed the data.

## Conflict of Interest Statement

LL is a co-founder of Synapse Medicine. The remaining authors declare that the research was conducted in the absence of any commercial or financial relationships that could be construed as a potential conflict of interest.

## Footnotes

1 http://ansm.sante.fr/Dossiers/Interactions-medicamenteuses/Interactions-medicamenteuses (accessed Aug 2018).

2 https://github.com/scossin/IMthesaurusANSM (accessed Aug 2018).

3 https://www.insee.fr/fr/statistiques/serie/000436387 (accessed Aug 2018).

4 Boston University School of Public Health: http://sphweb.bumc.bu.edu/otlt/MPH-Modules/BS/BS704_Confidence_Intervals/BS704_Confidence_Intervals8.html (accessed Dec 2018).

5 FASS Allmänhet – Startsida: https://www.fass.se/LIF/startpage

## Abbreviations

CCs: contraindicated codispensings
CDI Codispensings: codispensings contraindicated and discommended because of interactions
DCs: discommended codispensings
DDIs: drug-drug interactions
DIPS-score: drug interaction prevalence study-score
EGB: *Echantillon Généraliste des Bénéficiaires*
